# Supplementary material for: Effects of early adversity on the brain: Larger-volume anterior cingulate cortex in AIDS orphans
Source: PLoS One. 2019 Jan 14;14(1):e0210489. doi: 10.1371/journal.pone.0210489 (PMC6331092; doi:10.1371/journal.pone.0210489)
Supplement: S1 File — (DOC) [file pone.0210489.s001.doc]

**Title:** Effects of early adversity on the brain: Larger-volume anterior cingulate cortex in AIDS orphans

**Abstract**

Multiple studies have revealed that adolescent AIDS orphans have more psychosocial problems than healthy adolescents. However, little is known about whether and how the brain structures of adolescent AIDS orphans differ from those of healthy adolescents. Here, we used magnetic resonance imaging to compare adolescent AIDS orphans reared in institutions (*N* = 20) with a sex- and age-matched group of healthy adolescents reared in families (*N* = 20) in China using a voxel-based morphometry analysis. First, we found that both total gray- and white-matter volumes did not differ between groups. Second, after correcting for age, sex, and total gray-matter volume, the AIDS orphan group demonstrated smaller hippocampal volumes, larger anterior cingulate cortex (ACC) volumes, and no differences in the amygdala. Third, a whole-brain analysis identified higher gray-matter volume of the ACC in the AIDS orphan group than in the control group. The preliminary findings of this study highlight the need for future research to confirm the sensitivity of the hippocampus and ACC to early adversity.

***Key words***: early adversity; brain volume; hippocampus; anterior cingulate cortex

**Introduction**

An estimated 17.8 million AIDS orphans < 18 years old have lost either or both parents to AIDS , a population that was expected to increase to 25 million by 2015 . AIDS orphans experience many severe early adversities such as maternal deprivation , poverty , stigma , and multiple losses and erosion of family support . As a result, adolescent AIDS orphans have more psychosocial and behavioral problems than do healthy adolescents . For example, a study with a large sample size in China shows that AIDS orphans demonstrated poorer psychological adjustment than those from HIV-free families in the same rural community . Once adopted by institutions, AIDS orphans are more vulnerable and potentially at higher risk of lower degrees of life improvement and life satisfaction and more psychopathologies than are noninstitutionalized children.

In short, previous studies suggested that adolescent AIDS orphans, especially those reared in institutions, suffer more psychosocial and behavioral problems than healthy adolescents. However, very little is known about whether and how AIDS orphans differ from healthy adolescents at the neurobiological level. In particular, we aimed to identify the differences in neural structure between AIDS orphans and healthy children to determine how early adversity influences subsequent brain and behavioral development and develop effective intervention strategies to protect their health.

**Effect of early adversity on neural structure**

Several decades of neuroimaging studies have indicated that total gray- and white-matter volumes, the structures of the prefrontal cortex (PFC), hippocampus, and amygdala are highly sensitive to early stressful events, such as poverty , maternal deprivation , early exposure to institutionalization , and maltreatment . Therefore, AIDS orphans are predicted to show differences in these areas when compared to healthy adolescents.

Previous studies showed that gray- and white-matter volumes can be affected by institutional rearing experiences . For example, in English and Romanian Adoptees studies, Mehta et al. (2009) reported smaller gray- and white-matter volumes for 14 previously institutionalized adolescents (16 years old) relative to 11 never-institutionalized adoptees from the United Kingdom. Meanwhile, Sheridan et al. (2012) showed lower white-matter volumes among children who remained in institutional care than did children randomized to an improved rearing environment.

Multiple studies have reported that the PFC, which is responsible for temporal sequencing of goal-directed behaviors, is highly sensitive to early adversity . However, there are mixed findings from studies comparing the PFC volumes of children with early adversity to those of healthy children . Some studies have reported that regions of the PFC in institutionalized adolescents showed lower cortical thickness and volume than did those of community control subjects. The two most recent studies on maltreatment-related post-traumatic stress disorder (PTSD) observed larger gray-matter volumes of the middle-inferior and ventral regions of the PFC in the clinical groups .

Early adverse experiences are thought to influence the structure of the hippocampus and amygdala, two critical neural substrates that regulate stress responses, but the results are still mixed. For example, previous studies showed that poor children have smaller volumes of the hippocampus and amygdala . Meanwhile, maternal deprivation is correlated with smaller hippocampal volume , and smaller hippocampal volumes were found in later-adopted children relative to non-adopted controls . However, Mehta et al. (2009) failed to detect differences in hippocampal volumes in children with a history of institutional care. Moreover, institutionalized children reportedly showed higher amygdala volumes than did non-adopted adolescents , but larger amygdala volumes were not detected by Hodel et al. (2015), whose study focused on post-institutionalized children.

In sum, previous studies showed that early adversity was associated with differences in the volumes of the white and gray matter, PFC, hippocampus, and amygdala . However, the findings across studies regarding the specific regions affected that may be resulted by differences in sample composition are not entirely consistent . Institutional rearing of AIDS orphans represents a severe form of early psychological and physical neglect and would serve as a model system for understanding how early experiences impact brain and behavioral development.

**Current study**

The aim of the present study was to determine whether and how the brain structures of AIDS orphans differ from those of healthy adolescents. First, we investigated whether institutionally reared AIDS orphans had smaller cortical gray- and white-matter volumes relative to healthy adolescents. Second, regions of interest (ROI) analyses were performed to examine whether and how the gray-matter volumes of the PFC, hippocampus, and amygdala differ between AIDS orphans and healthy adolescents in. Third, a whole-brain analysis was performed to confirm the results of ROI analyses and determine which regions differed the most between the AIDS orphans and the controls.

**Methods**

**Subjects**

Twenty AIDS orphans (age, 15.75 ± 1.97 years; sex, 10 male, 10 female) were recruited from an orphanage located in Henan Province of China, an area with a high HIV/AIDS prevalence due to unsanitary commercial blood collections performed between the late 1980s and middle 1990s. They had lived in an orphanage a mean 5.08 ± 1.83, and 90% of them had been transferred from another orphanage (especially AIDS orphans attending primary school) to their current orphanage (especially older AIDS orphans). Another 20 school-aged adolescents (mean age, 15.85 ± 2.35; sex, 12 male, 8 female) were recruited from Beijing as the control group. No significant group differences were identified on age (*t* = 0.13; *p* = 0.90) and gender (*χ^2^* = 0.40; *p* = 0.53). Signed informed consent was obtained from the AIDS orphans and their legal guardians (their teachers in the orphanage) before the data collection. Meanwhile, written informed consent was obtained from all healthy adolescents and their parents. The magnetic resonance imaging (MRI) protocol and consent procedure were approved by the Institutional Review Board of Beijing Normal University.

**MRI data acquisition**

Participants were scanned in a Siemens 3T scanner (MAGENTOM Trio, a Tim system) with a 12-channel phased-array head coil at the BNU Imaging Center for Brain Research, Beijing, China. The whole-brain structural images were acquired using a 3D magnetization prepared rapid gradient echo (MP-RAGE) T1-weighted sequence (TR/TE/TI = 2530/3.39/1100 ms; flip angle = 7 degrees; field of view, 256 × 256 mm). One hundred and twenty-eight contiguous sagittal slices were acquired with 161-mm in-plane resolution and 1.33-mm slice thickness for whole-brain coverage.

**Voxel-based morphometry analysis**

Voxel-based morphometry (VBM) is a fully automatic technique which allows an objective analysis of anatomical differences between groups across the entire brain, and it has been widely used to quantify structural brain changes associated with neuropsychiatric pathologies such as psychiatric disorders and depression . Hence, VBM was employed to quantify gray-matter volume in each voxel across the entire brain (Ashburner and Friston, 2000). Specifically, VBM was performed using SPM8 ([Statistical Parametric Mapping](http://www.sciencedirect.com/science/article/pii/S0165032713001833#NEU11149) 8; Wellcome Department of Imaging Neuroscience, University College, London, UK) and DARTEL (Wellcome Department of Imaging Neuroscience) running under MATLAB 2010 (The Mathworks, Sherborn, MA, USA). First, image quality was assessed by visual inspection, and no excessive scanner artifact was found. Second, the origin of the brain was manually set to the anterior commissure for each participant. Third, images were segmented into four distinct tissue classes: gray matter, white matter, cerebrospinal fluid, and everything else (e.g., skull and scalp) using a unified segmentation approach . Fourth, the segmented gray-matter images were rigidly aligned and resampled to 2 × 2 × 2 mm. Fifth, the images were nonlinearly registered with DARTEL, which involves iteratively computing a study-specific template based on the tissue maps from all participants and then warping all participants’ gray-matter images into the generated template to increasingly improve the alignment (Ashburner, 2007). Sixth, the gray-matter images were normalized to standard Montreal Neurological Institute space, and the gray-matter voxel values were modulated by multiplying the Jacobian determinants derived from the registration to preserve the volume of tissue from each structure . The modulated gray-matter images were then smoothed with an 8-mm full width at half maximum isotropic Gaussian kernel. Finally, to exclude noisy voxels, the modulated images were masked using an absolute masking with a threshold of 0.2. The masked modulated gray-matter images were used in the further statistical analyses.

**MRI data analysis**

To fully investigate the neuroanatomical differences between the AIDS orphans and the control group, the current analysis started with an independent-sample *t*-test to compare the differences in the total white- and gray-matter volumes. Mean total gray- and white-matter volume were extracted for each participant using FreeROI (<http://freeroi.brainactivityatlas.org/>).

Second, ROI analyses were conducted to examine whether and how AIDS orphans differed from healthy adolescents in the PFC, hippocampus, and amygdala, which were hypothesized to be sensitive to early adversity based on previous studies (e.g., Mehta et al., 2009; Sheridan et al., 2012). All of the ROI masks were derived from Harvard-Oxford subcortical structural atlas available with FSL 4.1 (FMRIB, Oxford, UK; http://www.fmrib.ox.a-c.uk/fsl) with the threshold set at 50%. Specifically, the PFC consisted of four subdivisions: middle frontal gyrus (MFG), inferior frontal gyrus (IFG), anterior cingulate cortex (ACC), and the orbital frontal cortex (OFC) . The total amygdala and hippocampus volumes were obtained by adding right and left hemisphere. The independent-sample *t*-test was performed to identify differences in the gray-matter volumes of the PFC, amygdala, and hippocampus between AIDS orphans and healthy adolescents.

Third, to confirm the results of ROI analysis and determine which region differed the greatest between the AIDS orphans and controls, we further adopted a whole-brain VBM analysis. Total gray-matter volume, age, and sex were controlled for as confounding covariates. Through the analysis, we first identified clusters of contiguous voxels showing significant differences (*p* < 0.005, uncorrected) and then tested these clusters using cluster-based correction in the entire brain. In the whole-brain correction, the minimum cluster size above which the probability of type I error was <0.05 was determined by the cluster program in FSL using the Gaussian Random Field theory. Local peak voxels were extracted using FreeROI.

**Results**

All analyses first reported the unadjusted comparison results of neural structure between AIDS orphans and the control group followed by the results adjusted for covariates such as age and sex. Analyses of the PFC, hippocampus, and amygdala were additionally adjusted for total gray-matter volume.

**Total gray-matter volume**

The independent-sample *t*-test identified no significant differences in the total gray-matter volume between the AIDS orphans and the control group (*t* = -0.78, *p* = 0.439). After the addition of age and sex as confounding covariates, the two groups still showed no significant difference in total gray-matter volume (*t* = -0.54, *p* = 0.591).

**Total white-matter volume**

The total white-matter volume did not differ significantly between AIDS orphans and adolescents in the control group (*t* = -0.49, *p* = 0.625). After the adjustment for age and sex, no significant group difference was identified (*t* = -0.23, *p* = 0.822).

**Prefrontal cortex**

The PFC was subdivided into the MFG, IFG, ACC, and OFC according to previous studies . The ROI analysis showed no group difference in the gray-matter volume of PFC (*t* = 0.07, *p* = 0.947), as well as of all the sub-regions (MFG: *t* = -1.14, *p* = 0.261; IFG pars opercularis: *t* = -0.49, *p* = 0.629; IFG pars triangularis: *t* = -0.68, *p* = 0.500; ACC: *t* = 1.17, *p* = 0.250; OFC: *t* = 0.61, *p* = 0.544) (Table 1). When controlled for age, sex, and total gray-matter volume, significant group differences were identified in the PFC (*t* = 2.49, *p* = 0.017) and ACC (*t* = 3.21, *p* = 0.003), while a marginal difference was found in OFC (*t* = 2.01, *p* = 0.052). These findings indicated that AIDS orphans have larger gray-matter volume in PFC as well as its sub-regions like the ACC and OFC than adolescents in the control group (Figure 1).

Insert Table 1 and Figure 1 here

**Hippocampus**

The gray-matter volumes of the hippocampus (total hippocampus: *t* = -2.06, *p* = 0.046; left hippocampus: *t* = -2.07, *p* = 0.047; right hippocampus: *t* = -2.00, *p* = 0.053) differed between the AIDS orphans and the control group (Table 1). After the control for age, sex, and total gray-matter volume, AIDS orphans still demonstrated significantly smaller gray-matter volume in hippocampus (total hippocampus: *t* = -2.16, *p* = 0.037; left hippocampus: *t* = -2.14, *p* = 0.039; right hippocampus: *t* = -2.01, *p* = 0.052) than adolescents in the control group (Figure 2).

Insert Figure 2 here

**Amygdala**

No significant group difference was found in the volume of the amygdala (total amygdala: *t* = -1.38, *p* = 0.174; left amygdala: *t* = -1.67, *p* = 0.103; right amygdala: *t* = -1.11, *p* = 0.273) (Table 1). After the control for age, sex, and total gray-matter volume, AIDS orphans and adolescents in the control group still showed no difference in gray-matter volume of the amygdala (amygdala: *t* = -1.26, *p* = 0.217; left amygdala: *t* = -1.81, *p* = 0.080; right amygdala: *t* = -0.75, *p* = 0.461).

**Whole-brain VBM analysis**

To confirm the ROI analysis results and determine which region differed the most between the AIDS orphans and the controls, a whole-brain VBM analysis was conducted. The results suggested that, when compared with adolescents in the control group, AIDS orphans reared in the orphanage exhibited significantly larger gray-matter volumes in the ACC (minimum coordinate: 2, -4, 44; cluster size: 1823; *p* = 0.005, cluster-based correction) (Table 2, Figure 3). This finding was consistent with the results of the ROI analysis conducted in ACC and further indicated that ACC was the largest different region between the AIDS orphans reared in an orphanage and the healthy adolescents.

Insert Table 2 and Figure 3 here

**Discussion**

In this first volumetric study of the brain in AIDS orphans reared in institutions, we found substantial support for the hypothesis that institutionalized AIDS orphans have structural brain alternations in a previously predicted ROI (PFC and hippocampus) based on previous studies . Specifically, we found no group differences in the volumes of total gray and white matter between the AIDS orphans and the control group. Second, after the correction for age, sex, and total gray-matter volume, ROI analysis showed that AIDS orphans developed smaller hippocampal but larger ACC volumes than healthy adolescents, but no differences was observed in amygdala volumes. Third, the whole-brain analysis showed that the volume difference of the ACC was the largest between the AIDS orphans and the controls. Determining the differences in neural structures between AIDS orphans reared in institutions and those raised in families is an important step in increasing our understanding the mechanisms by which adverse experiences lead to behavioral and psychological deficits and ultimately for developing clinical interventions.

Despite the obvious paucity of structural imaging studies of AIDS orphans reared in institutions, our findings are largely consistent with those of previous studies of individuals suffering from early adversity like poverty, maternal deprivation, and maltreatment, especially with respect to significant reductions in hippocampal volumes (adjusted and unadjusted) . The reason that AIDS orphans have smaller hippocampi may be that the hippocampus contains a high density of glucocorticoid receptors, so it appears particularly susceptible to the deleterious effect of chronic stress created by early adversity associated with the AIDS diagnosis and institutional rearing, such as stigma and poverty . Our findings of AIDS orphans reared in institutions replicated previous studies that observed decreased gray-matter volume of the hippocampus in children exposed to other early adversities and suggested that different types of adversity, such as stigma, maternal deprivation, and poverty, might share a common neural pathway when exerting influence on adolescents’ health.

Although our findings were inconsistent with those of previous MRI studies that showed reductions of PFC volumes in institutionalized children , we found that the largest increase in volume occurs in the PFC (especially the ACC). For example, Hodel et al. (2015) showed that 12–14-year-old post-institutionalized children have significant reductions in PFC volume relative to the comparison group reared in families in the United States. In contrast, another study using VBM found that children (aged 7–14) with a history of interpersonal trauma have larger gray-matter volume of the PFC than do control subjects . Why AIDS orphans developed larger ACC volumes than healthy adolescents is not yet clear, but one possibility is that larger ACC volumes might contribute to AIDS orphans’ resilience in adversity. Resilience refers to one’s capability to adapt flexibly, persistently, and resourcefully to stressful situations . A recent study revealed that the ACC involves the cognitive regulation to change one’s emotional response to stressful events that might promote people’s resilience to adversity. In particular, there is evidence that people who experience trauma but do not develop PTSD (more resilient people) show greater ACC activity , while PTSD patients with larger ACC volume achieve greater treatment gains during psychological therapy . Furthermore, our findings showed that the gray-matter volumes in the dorsal and ventral ACC differed the greatest between AIDS orphans and healthy adolescents. Considering that neuroimaging studies divide the ACC into “cognitive/dorsal” and “affective/rostral” subdivisions , the current finding suggests that a larger ACC might contribute to promoting resilience through better cognitive and affective processing of information. To validate this assumption, future studies are needed to investigate whether a larger ACC might guarantee AIDS orphans enhanced resilience to ameliorate their psychological distress. Furthermore, the integration of resilience-based intervention and neuroscience would help uncover which factors played central roles (e.g., self-esteem, optimism, or social support) in the resilience-based interventions targeting adolescents affected by HIV/AIDS and the development of more targeted clinical interventions .

In contrast with two previous studies of institutionalized children , we found no evidence of altered amygdala volume between AIDS orphans and healthy adolescents, which is consistent with the findings of Hodel et al. (2015). One possibility is that the amygdala is a small region of the brain and particularly difficult to segment using VBM ; further improvements in amygdala segmentation may contribute to determining the influences of early adversity on the amygdala structure . Meanwhile, we found no differences in total gray- and white-matter in AIDS orphans relative to healthy adolescents, which was also inconsistent with previous studies (Mehta et al., 2009; Sheridan et al., 2012). One plausible explanation is that the heightened resilience associated with a larger ACC might the gray and white matter of AIDS orphans from the harm of early adversity.

In summary, this study is the first to demonstrate that AIDS orphans reared in institutions developed smaller hippocampal volumes and larger ACC volumes than did healthy adolescents. We acknowledge several limitations of the current study that make its replication in a larger sample set desirable. Considering that the subjects of the orphan and control group were enrolled from different cities in China and had different socioeconomic backgrounds, socioeconomic status is a potential confounder in the current study. To confirm the current findings, it is better to compare AIDS orphans with healthy adolescents from the same area (such as Henan province) in future studies. Meanwhile, because of the current small sample of subjects and our inability to gather the participants’ histories of early stressful events, we cannot determine which adversity led to the neural differences between AIDS orphans and healthy adolescents. Therefore, a future strategy will explore whether specific brain changes in AIDS orphans are related to specific types of adversity. For example, whether the experience of being orphaned by AIDS or the institutional rearing led to differential brain alterations remained to be answered. Another limitation to the generalizability of our results is that our sample included only Chinese participants. Moreover, the sample contained very few subjects. The generalizability of our findings is limited until we learn whether these results are robust across samples with different races and ages. Moreover, fundamental questions regarding the effects of mediators or moderators on brain volume differences in AIDS orphans need to be addressed in future studies. There is also a need to study the differential roles of the hippocampus and PFC in predicting behavioral differences between AIDS orphans and healthy adolescents.

Despite these shortcomings, the finding that AIDS orphanhood was related to a smaller hippocampus and a larger PFC has important research and clinical implications. First, structural changes in certain brain regions may be potential biomarkers for outlining the influence of AIDS orphanhood imposed on children, which reflects individuals’ vulnerability to early traumatic experiences and a series of unfolding stressors. Second, if an intervention could normalize the brain anatomical differences found in AIDS orphans, it may provide valuable clues about how an individual responds to the intervention as well as future research on intervention targeting this population. Based on the current findings, interventions focused on promoting AIDS orphans’ resilience may be applicable to mitigate the impacts of early adversity on neurobehavioral development.
